# The neutrophil to lymphocyte ratio and serum albumin as predictors of acute kidney injury after coronary artery bypass grafting

**DOI:** 10.1038/s41598-022-19772-7

**Published:** 2022-09-14

**Authors:** Masashi Ishikawa, Masae Iwasaki, Dai Namizato, Makiko Yamamoto, Tomonori Morita, Yosuke Ishii, Atsuhiro Sakamoto

**Affiliations:** 1 Department of Anesthesiology and Pain Medicine, Graduate School of Medicine, Nippon Medical School, 1-1-5 Sendagi, Bunkyo-ku, Tokyo, 113-8603 Japan; 2 Department of Cardiovascular Surgery, Nippon Medical School Hospital, 1-1-5Bunkyo-ku, Sendagi, 113-8603 Japan

**Keywords:** Medical research, Risk factors

## Abstract

Postoperative acute kidney injury (AKI) is a highly prevalent and serious complication after cardiac surgery. The aim of this study is to identify the predictors of AKI and the cut-off values after isolated off-pump coronary artery bypass grafting (OPCAB). A total of 329 adult patients, who underwent isolated OPCAB between December 2008 and February 2021, were retrospectively analyzed. The patients were divided into three groups: non-AKI, early AKI and late AKI groups. The early AKI group or the late AKI group were defined as ‘having AKI that occurred before or after 48 h postoperatively’, respectively. Multivariate logistic regression analysis was performed to identify the predictors of AKI. Receiver operating characteristic (ROC) curve analysis was used to evaluate the cutoff value, the sensitivity, and the specificity of the predictors. On the multivariate analysis, the emergency surgery, the preoperative serum albumin, and the postoperative day 1 neutrophil to lymphocyte ratio (NL ratio) were identified as the independent predictors of AKI. However, neither albumin nor the NL ratio predicted late AKI. The present study showed the preoperative albumin and the postoperative day 1 NL ratio were the robust and independent predictors of postoperative early AKI in isolated OPCAB.

## Introduction

Postoperative acute kidney injury (AKI) is a highly prevalent and serious complication after cardiac surgery. The incidence of AKI after cardiac surgery ranges from 13 to 43%, and AKI increases short- and long-term morbidity and mortality^1–3^. To predict postoperative AKI, numerous studies have identified several risk factors, including older age, body mass index, the duration of cardiopulmonary bypass (CPB), preoperative hypertension, the reduced left ventricular ejection fraction, and the preoperative impaired renal function^4^. The frequency of renal function evaluation after discharge from the intensive care unit decreases, making it difficult to detect and treat AKI early. Thus, the prediction of AKI, especially late-onset AKI, is very important for clinicians. Early appropriate prediction and focused monitoring for patients at high risk of AKI are paramount. C-reactive protein (CRP) is a representative marker of the acute proinflammatory process^5^ and one of the predictors of AKI^6, 7^. The postoperative day 1 neutrophil to lymphocyte ratio (NL ratio) is associated with postoperative AKI in cardiovascular surgery with CPB^8^. The cardiac biomarker, creatine kinase-MB (CK-MB), is also associated with AKI and mortality risk after cardiac surgery^9,10^. Moreover, an association between hypoalbuminemia and AKI has been reported in contrast-induced nephropathy^11^ and postoperative AKI in coronary artery bypass grafting (CABG)^12^. Although some previous reports have especially focused on inflammatory or cardiac markers as predictors of AKI after cardiac surgery^9,13^, the predictors of AKI in isolated off-pump CABG (OPCAB) have not been elucidated.

The aim of this study was to identify the predictors and cut-off values of AKI after isolated OPCAB. We evaluated whether early or late AKI following isolated OPCAB could be predicted by previously identified predictors of AKI following other cardiac procedures, the preoperative hemoglobin value, albumin, the NL ratio, CK-MB, and CRP on postoperative day (POD) 1.

## Results

The total 329 patients in the present study, mean age 70.9 ± 8.9 years old, included 260 men (79.0%). AKI developed in 67 patients (20.4%); 46 patients (68.7%) had stage 1, 11 (16.4%) patients had stage 2, and 10 patients (14.9%) had stage 3 (Table 1). Among 67 patients with AKI, 44 patients (65.7%) had early AKI, and 23 patients (34.3%) had late AKI. Postoperative renal replacement therapy was required for 10 patients (14.9%). The demographic and intraoperative data of the entire cohort were shown in Table 2.

**Table 1:** Frequency of postoperative acute kidney injury.

	AKI	Early AKI	Late AKI
AKI	67	44	23
Stage 1	46 (68.7%)	29 (65.9%)	17 (73.9%)
Stage 2	11 (16.4%)	7 (15.9%)	4 (17.4%)
Stage 3	10 (14.9%)	8 (18.2%)	2 (8.7%)

AKI: acute kidney injury.

**Table 2:** Patients’ baseline characteristics.

Variable	All cases	Non-AKI	AKI	*P* value
Sex: male (%)	260 (79.0)	205 (78.2)	55 (82.1)	0.103
Age (y)	70.9 ± 8.9	70.5 ± 9.0	72.5 ± 8.3	0.948
BMI (kg/m^2^)	24.0 ± 3.2	24.0 ± 3.1	24.2 ± 3.8	0.759
Complication				
Hypertension	236 (7.17)	189 (72.1)	47 (70.1)	0.748
Atrial fibrillation	13 (4.0)	7 (2.7)	6 (9.0)	0.033
Heart failure	29 (8.8)	21 (8.0)	8 (11.9)	0.329
Mitral regurgitation	79 (24.0)	64 (24.4)	15 (22.4)	0.726
Tricuspid regurgitation	49 (14.9)	42 (16.0)	7 (10.4)	0.235
Aortic regurgitation	39 (11.9)	32 (12.2)	7 (10.4)	0.686
Aortic stenosis	9 (2.7)	6 (2.3)	3 (4.5)	0.357
Diabetes mellitus	164 (49.8)	134 (51.1)	30 (44.8)	0.352
Chronic kidney disease	140 (42.6)	102 (38.9)	38 (56.7)	0.009
Ejection fraction	55.7 ± 14.6	56.1 ± 14.4	54.1 ± 15.0	0.316
Emergency OPCAB	42 (12.8)	24 (9.2)	18 (26.9)	< 0.001
Operation time (min)	313.3 ± 82.2	315.6 ± 79.6	304.6 ± 91.8	0.332
Water balance (mL)	4,139.6 ± 1581.2	4,217.5 ± 1565.7	3,835.0 ± 1616.4	0.077
Urine (mL)	857.9 ± 747.6	918.4 ± 774.1	621.2 ± 580.0	0.004
Intraoperative blood loss (mL)	389.2 ± 279.6	370.9 ± 265.7	460.6 ± 320.8	0.019

AKI: acute kidney injury, BMI: body mass index, OPCAB: off-pump CABG.

Univariate analysis showed significant differences between the non-AKI group and the AKI group in the preoperative atrial fibrillation (non-AKI group vs AKI group, 2.7% vs 9.0%, *P* = 0.033), the preoperative chronic kidney disease (38.9% vs 56.7%, *P* = 0.009), emergency OPCAB (9.2% vs 26.9%, *P* < 0.001), intraoperative urine volume (918.4 ± 774.1 vs 621.2 ± 580.0, ml, *P* = 0.004), intraoperative blood loss (370.9 ± 265.7 vs 460.6 ± 320.8, ml, *P* = 0.019), the preoperative serum albumin (3.9 ± 0.5 vs 3.6 ± 0.6, g/dl, *P* < 0.001), and the NL ratio (6.7 ± 5.1 vs 9.7 ± 5.4, postoperative day 1, *P* < 0.001, Tables 2 and 3). With the multivariate analysis, emergency OPCAB, the preoperative albumin, and the NL ratio were identified as independent predictors of AKI (emergency OPCAB: odds ratio (OR): 3.06, 95% confidence interval (CI): 1.45–6.41, *P* = 0.004; the preoperative albumin: OR: 0.47, 95% CI: 0.28–0.79, *P* = 0.004; the NL ratio: OR: 1.08, 95% CI: 1.02–1.15, *P* = 0.007, Table 4). Although both albumin and the NL ratio were significantly changed in the AKI (non-AKI group vs AKI group, albumin: 3.9 ± 0.5 vs 3.6 ± 0.6, *P* = 0.002, NL ratio: 6.7 ± 5.1 vs 9.7 ± 5.4, *P* < 0.001) and early AKI group (non-AKI group vs early AKI group, albumin: 3.9 ± 0.5 vs 3.5 ± 0.6, *P* < 0.001, NL ratio: 6.7 ± 5.1 vs 11.1 ± 5.8, *P* < 0.001), they did not predict late AKI occurrence (non-AKI group vs late AKI group, albumin: 3.9 ± 0.5 vs 3.8 ± 0.5, *P* = 0.906, NL ratio: 6.7 ± 5.1 vs 7.1 ± 3.2, *P* = 0.991) (Table 5). The results of multiple regression analysis were y = 0.626 + 0.012 × (operation time, min)  − 0.001 × (Water balance, ml) + 0.003 × (bleeding, ml)  − 1.191 × (chronic heart failure) − 0.838 × (chronic kidney disease) for NL ratio, and y = 4.269–0.012 × (age, year) + 0.108 × (chronic heart failure) for albumin. These results indicate the slight confounding effect, but these conditions seem less impacts in the clinical situation.

**Table 3:** Laboratory findings.

Variable	All cases	Non-AKI	AKI	*P* value
Hemoglobin	12.6 ± 1.0	12.7 ± 2.0	12.2 ± 2.0	0.058
Albumin	3.8 ± 0.6	3.9 ± 0.5	3.6 ± 0.6	< 0.001
Neutrophil to lymphocyte ratio	7.3 ± 5.3	6.7 ± 5.1	9.7 ± 5.4	< 0.001
CK-MB	14.3 ± 20.8	13.4 ± 18.5	18.3 ± 28.3	0.087
CRP	7.1 ± 3.6	6.9 ± 3.4	7.7 ± 4.5	0.136

AKI: acute kidney injury, CK-MB: creatine kinase MB, CRP: C-reactive protein.

**Table 4:** Risk factors for acute kidney injury occurrence on multivariate analyses.

Variable	Odds ratio	95% CI	*P* value
Atrial fibrillation	2.62	0.75–8.81	0.128
Chronic kidney disease	1.74	0.955–3.22	0.071
Emergency	3.06	1.45–6.41	0.004
Intraoperative blood loss	1	0.99–1.01	0.149
Albumin	0.47	0.28–0.79	0.004
Neutrophil to lymphocyte ratio	1.08	1.02–1.15	0.007

CI: confidence interval.

**Table 5:** Laboratory findings in each group.

Variable	Non-AKI	AKI	Early AKI	Late AKI	P value		
					AKI	Early AKI	Late AKI
Albumin	3.9 ± 0.5	3.6 ± 0.6	3.5 ± 0.6	3.8 ± 0.5	0.002	< 0.001	0.906
Neutrophil to lymphocyte ratio	6.7 ± 5.1	9.7 ± 5.4	11.1 ± 5.8	7.1 ± 3.2	< 0.001	< 0.001	0.991

P value: vs non-AKI.

AKI: acute kidney injury.

**Figure 1:**
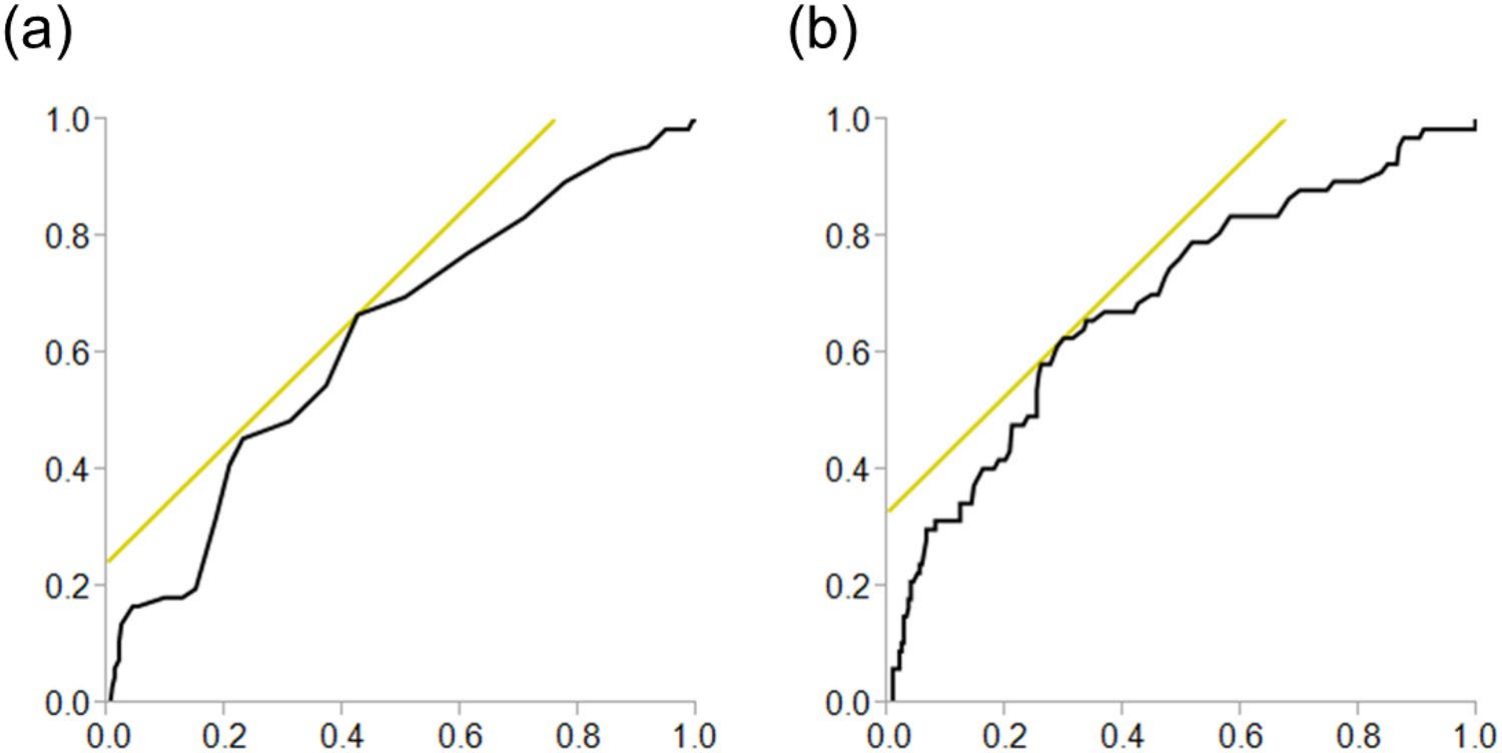
Receiver operating characteristic curves for albumin and the neutrophil to lymphocyte ratio for predicting acute kidney injury. (**a**) albumin, (**b**) neutrophil to lymphocyte ratio.

**Figure 2:**
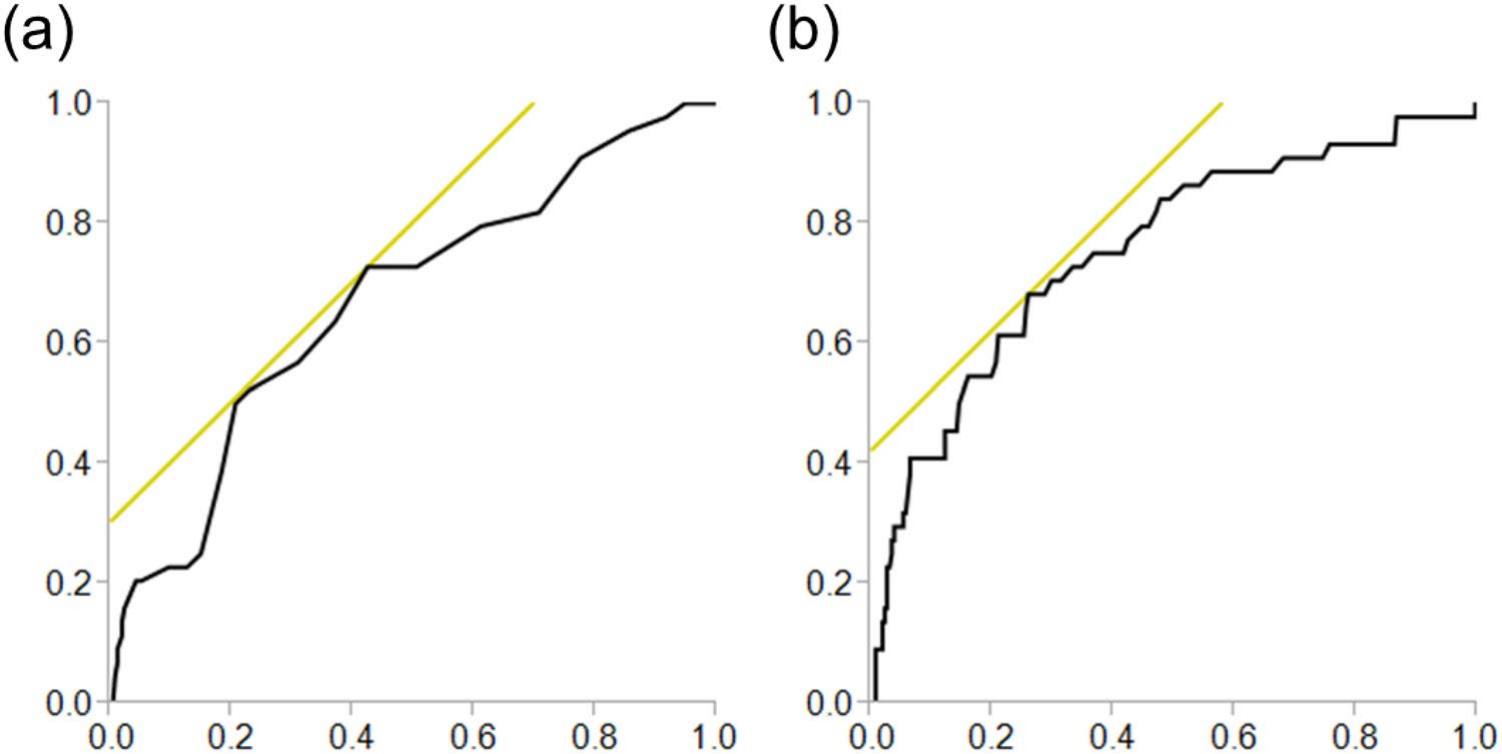
Receiver operating characteristic curves for albumin and the neutrophil to lymphocyte ratio for predicting early acute kidney injury. (**a**) albumin, (**b**) neutrophil to lymphocyte ratio.

The receiver operating characteristic (ROC) curve analyses of preoperative albumin and the NL ratio were summarized in Table 6 and Figs. 1 and 2. Areas under the curve (AUC) for albumin were 0.635 (*P* < 0.001) in the AKI group and 0.672 (*P* < 0.001) in the early AKI group. A threshold postoperative albumin value of 3.8 was associated with the occurrence of AKI with a sensitivity of 67% and specificity of 58%, and that of 3.8 was associated with the occurrence of early AKI with a sensitivity of 73% and specificity of 58%. On the other hand, AUC for NL ratio were 0.693 (*P* < 0.001) in the AKI group and 0.755 (*P* < 0.001) in the early AKI group. A threshold postoperative NL ratio value of 7.3 was associated with the occurrence of AKI with a sensitivity of 63% and specificity of 70%, and that of 7.8 was associated with the occurrence of early AKI with a sensitivity of 68% and specificity of 74%.

**Table 6:** Data from receiver operating characteristic curves for albumin and the neutrophil to lymphocyte ratio for predicting acute kidney injury.

	Cutoff	AUC	Sensitivity	Specificity	*P* value
Albumin					
AKI	3.8	0.635	67%	58%	< 0.001
Early AKI	3.8	0.672	73%	58%	< 0.001
Neutrophil to lymphocyte ratio					
AKI	7.3	0.693	63%	70%	< 0.001
Early AKI	7.8	0.755	68%	74%	< 0.001

AKI: acute kidney injury, AUC: area under the curve.

## Discussion

A total of 329 patients who underwent OPCAB were analyzed, and the incidence of AKI was 20.4%. In this study, the Kidney Disease: Improving Global Outcome (KDIGO) criteria were used to define AKI. The diagnostic criteria for AKI have been updated to the KDIGO from RIFLE or Acute Kidney Injury Network (AKIN). In some previous reports^1,2^, the RIFLE criteria or AKIN criteria were used for AKI diagnosis. One of the differences between the KDIGO criteria and other AKI guidelines, the RIFLE and the AKIN criteria, is the duration of AKI. The KDIGO criteria defines AKI as 1 week duration, while other criteria define AKI as 48-h duration. Chen et.al.^4^ revealed the predictive factors of postoperative AKI in the isolated CAB patients with the KDIGO criteria. However, late AKI may be influenced by many factors such as postoperative management, hemodynamic condition, medication, infection and bleeding. Late AKI accounted for 34.3% of all AKI. In the present study, the preoperative albumin and the NL ratio were found to be predictors of AKI and early AKI, and the sensitivity of the NL ratio was higher than that of preoperative albumin. However, these two factors were not significant predictors of late AKI.

AKI is a common complication after CABG that is associated with the postoperative mortality^14,15^. Most cases of AKI after cardiac surgery are temporary and reversible. Patients with mild AKI are usually responsive to prompt medical interventions, such as avoiding additional renal insults and optimizing volume status. Medical therapy can prevent the progression of perioperative AKI and improve postoperative outcomes such as mortality^16–18^. Early detection of AKI may contribute to improving patients’ outcomes^19^. Therefore, the reliable predictors of AKI that show changes earlier than decreased urine volume and elevated serum creatinine are needed.

The pathophysiology of AKI after cardiac surgery is multifactorial, including inflammation, ischemia–reperfusion injury, operative trauma, neurohormonal activation, metabolic changes, and oxidative stress^20–22^. Cardiovascular surgery causes an activation of the inflammatory cascade because of the preoperative patients’ disease and surgical trauma. Although cytokines have the role as mediators of immunological responses to surgery, these biomarkers are not quickly available. Some cytokines are commonly well known their pro-inflammatory and anti-inflammatory functions regulating lymphocyte activity^23^. Neutrophils play an important role in inflammation, and a reduced lymphocyte count reflects physiological stress^24,25^. Dynamic change in the NL ratio is attributed to systemic inflammation. High NL ratio significantly increased the risk of mortality, post-operative re-intubation, limb amputation, and postoperative atrial fibrillation after cardiovascular operations^26^ and OPCAB^24,27^. Moreover, postoperative NL ratio was also independently associated with postoperative AKI following isolated CABG, because elevated NL ratio suggest an association between inflammation and renal failure^28^. Therefore, NL ratio could contribute to early identification of patients at high risk for periprocedural adverse events^29^. The relationship between AKI and the NL ratio in patients undergoing isolated OPCAB has not been studied sufficiently. One meta-analysis of the NL ratio for the prediction of AKI estimated an area under the curve of 0.65 in a cardiac surgery subgroup^30^. In the present study, ROC curve analysis yielded a similar result for the NL ratio (AUC: 0.693) with a cutoff value of 7.3 (63% sensitivity, 70% specificity) (Fig. 1). In addition, the NL ratio can also predict early AKI with a cutoff value of 7.8 (AUC: 0.755, sensitivity 68%, specificity 74%) (Fig. 2).

Hypoalbuminemia (often defined as < 3.5–4.0 g/dL) is a well-established risk factor for morbidity and mortality^31,32^. An association between hypoalbuminemia and AKI has been reported in contrast-induced nephropathy^11^ and postoperative AKI following cardiac surgery^12,33^. In patients with hypoalbuminemia, the glycocalyx might be compromised, leading to the loss of oncotic pressure gradients and barrier function, the fluid leakage into the tissue, and the microvascular flow alterations^34,35^. The serum albumin protects renal function by maintaining the oncotic pressure, which augments the intravascular volume^36^, maintains renal perfusion, and improves glomerular filtration^37^. Albumin also limits tubular cell apoptosis as a scavenger of radical oxygen species and roles as an anti-inflammation effector^38,39^. The reported cutoff values of albumin vary between previous studies due to the study populations and types of surgery. In a study with non-cardiac surgery patients, the cutoff value of 3.75 g/dL had a sensitivity of 54% and specificity of 67%; meanwhile, among patients undergoing brain tumor surgery, the cutoff value of 3.8 g/dL had a similar sensitivity of 54%, but lower specificity of 27%^40,41^. Our ROC curve analysis indicated that, albumin with a cutoff value of 3.8 (AUC 0.635, sensitivity 67%, specificity 58%) was a valuable predictor of AKI in isolated OPCAB. Our analysis also showed that albumin could predict early AKI with a cutoff value of 3.8, not for late AKI.

The present study hypothesized that CK-MB and CRP could be the predictors of AKI in isolated OPCAB patients. CK-MB is a specific myocardial marker that increased by myocardial ischemia and hypotension. Therefore, postoperative CK-MB elevation suggests intraoperative myocardial damage. Perioperative cardiac biomarkers including CK-MB are associated with an increased mortality risk after CABG with CPB^10^. Moreover, cardio-renal syndrome is a well-known interdependency of cardiac and renal dysfunction in cardiac disease. Preoperative CK-MB was a strong and independent predictor of postoperative AKI in cardiac surgery^9^. CRP is a representative marker of acute inflammation and predicts the severity of acute infection and long-term morbidity or mortality^5,42^. A high CRP level is a biomarker of AKI or mortality in cardiac disease^6,42,43^ and post-CABG patients with CPB^7^. However, they did not predict postoperative AKI in the present study. They are associated with AKI indirectly through myocardial damage or inflammation. Although these factors were measured on POD1, no significant increases were observed. For predictors to be useful, they need to increase early, before AKI is established.

The present study had some limitations. Firstly, this study is a single-center, retrospective, observational study. A prospective, multicenter study is needed to substantiate its broad applicability. Secondary, the period of this study is a long-time span. The surgical and anesthetics technology may be improved during the study period. Finally, we didn’t clarify whether N/L ratio and albumin were correlated with other factors because of limited sample number. The potential correlation may cause bias.

In conclusion, this study showed that preoperative albumin and the POD1 NL ratio are robust and independent predictors of postoperative AKI in isolated OPCAB. Both markers can be easily measured in general ward and detect patients at high risk of AKI and early AKI with high sensitivity. The further investigation of the late AKI predictors should be needed in OPCAB patients.

## Methods

### Participants and data collection

After approval by the ethics committee of Nippon Medical School (No. 2021–192). This study was performed in accordance with relevant guidelines and regulations. 329 adult patients who underwent isolated OPCAB between December 2008 and February 2021 at Nippon Medical School Hospital were retrospectively analyzed. Patients who lacked data or needed preoperative renal replacement therapy or circulatory assist devices in the perioperative period were excluded. Informed consent was obtained from patients through an opt-out method.

The patients’ hospital records, which included patient demographic information (sex, age, body mass index (BMI), preoperative comorbidities, examination data), their surgical management (emergency, duration of surgery, intraoperative blood loss), anesthetic management (water balance, urine volume), and postoperative data (urine volume, laboratory data), were collected. The KDIGO criteria for AKI were used in the present study based on the change in serum creatinine levels (≥ 0.3 mg/dL within 48 h or ≥ 50% within 7 days) or urine output volume less than 0.5 mL/kg/hour for > 6 hours^44^. The patients were divided into three groups: non-AKI, early AKI and late AKI groups. The early or late AKI group were defined as having AKI that occurred before or after 48 h postoperatively, respectively. The predictors of AKI in preoperative laboratory data, hemoglobin and albumin, in postoperative laboratory data, NL ratio, CK-MB and CRP, were measured within 1 week before surgery and at the postoperative day 1, respectively. NL ratio was calculated by dividing the number of neutrophils to the number of lymphocytes.

### Anesthetic technique

All patients underwent general anesthesia with endotracheal intubation and ventilation. Anesthesia was maintained with sevoflurane, fentanyl, and rocuronium. An arterial blood catheter, transesophageal echocardiography, and a central venous catheter were routinely used. When transient hypoperfusion occurred during manipulation of the heart or anastomosis, systemic perfusion pressure was usually maintained by tilting the surgical table and administering intravenous fluids, followed by the administration of noradrenaline as a first-choice vasopressor. All patients were transferred to intensive care unit intubated postoperatively. They were extubated following normalization of orientation, hemodynamic and respiratory functions.

### Statistical analysis

Clinical data were recorded and tabulated using Excel software (Microsoft Corp, Redmond, WA, USA). All statistical analyses were performed using JMP version 11 software (SAS Institute Inc., Cary, NC, USA). The results are expressed as means ± SD or n (%). P values of 0.05 were considered significant. Continuous variables were compared between groups using the two-tailed unpaired Student’s *t-*test. For dichotomous variables, group differences were examined using Fisher’s exact test. The postoperative serum markers were compared among three groups with Tukey’s test. Multivariate logistic regression analysis was performed to identify the predictors of AKI with independent variables. The model was built using variables that demonstrated a *P* < 0.05 on univariate analysis, except for confounding factors. Significance within the model was evaluated by the likelihood ratio test, and the strength of the association of variables with AKI was estimated by calculating the OR and 95% CI. Multiple regression analysis was performed to evaluate the confounding factors of postoperative day 1 NL ratio and preoperative albumin value. ROC curve analysis was used to compute the cutoff value, sensitivity, and specificity of the predictors.

## Data Availability

All data generated or analyzed during this study are included in this published article. Anonymized data for the current study are available from the corresponding author upon reasonable request.
